# Cytotoxicity assessment of ZnO nanoparticles on human epidermal cells

**DOI:** 10.1186/1755-8166-7-S1-P81

**Published:** 2014-01-21

**Authors:** Pal Patel, Krupa Kansara, Darshini Shah, NV Srikanth Vallabani, Ritesh K Shukla, Sanjay Singh, Alok Dhawan, Ashutosh Kumar

**Affiliations:** 1 Institute of Life Sciences, School of Science and Technology, Ahmedabad University, Ahmedabad, Gujarat, India

## Background

Nanotechnology is growing rapidly worldwide and engineered nanoparticles have found tremendous applications in consumer and industrial products. Metal oxide nanoparticles (NPs), especially zinc oxide (ZnO), are widely used in cosmetics, catalysis, electronics, biosensors, medicine, paints, food packaging and imaging. As, the ZnO NPs have major application in cosmetics, their exposure will mainly be through the skin, which is the largest organ of the body and could serve as an important portal route for entry. Therefore, the present study was carried out to assess the cytotoxicity of ZnO NPs on human epidermal cells (A431).

## Materials and methods

The average hydrodynamic size, size distribution, zeta potential and stability of ZnO NPs were determined by dynamic light scattering (DLS). The detection for the internalization of ZnO NPs was carried out using flow cytometry. Cytotoxicity response was assessed by neutral red uptake (NRU) and MTT [3-(4, 5-dimethylthiazoyl-2-yl)-2, 5-diphenyltetrazolium bromide] assays. Further, to analyze the ability of ZnO NPs to induce the reactive oxygen species (ROS) generation and oxidative damage, 2, 7-dichlorofluorescein diacetate (DCFDA) dye was used. The effect of ZnO NPs in progression of cell cycle was also assessed using propidium iodide dye by flow cytometer.

## Results

The mean hydrodynamic diameter and zeta potential of ZnO NPs in DMEM with 10% FBS was 30.95 ± 3.72 nm and -12.8 ± 0.6 mV respectively. In flow cytometry, a concentration dependent significant (p<0.05) increase in the side scatter (SSC) intensity of treated cells was observed after 6 hr exposure. A significant (p<0.05) dose and time dependent cytotoxicity as evident from MTT and NRU assays was also observed. Additionally, a significant (p<0.05) induction in ROS generation was also observed in a dose dependent manner.

## Conclusion

Our study demonstrates that ZnO NPs induce cytotoxicity in human epidermal cells. Hence, proper precautions need to be taken while handling such NPs.

